# Exposure to Residential Green Space and Bone Mineral Density in Young Children

**DOI:** 10.1001/jamanetworkopen.2023.50214

**Published:** 2024-01-04

**Authors:** Hanne Sleurs, Ana Inês Silva, Esmée M. Bijnens, Yinthe Dockx, Martien Peusens, Leen Rasking, Michelle Plusquin, Tim S. Nawrot

**Affiliations:** 1 Centre for Environmental Sciences, Hasselt University, Hasselt, Belgium; 2 Department of Environmental Sciences, Faculty of Science, Open University, Heerlen, the Netherlands; 3 Department of Epidemiology and Public Health, Sciensano, Brussels, Belgium; 4 Department of Public Health and Primary Care, Leuven University, Leuven, Belgium

## Abstract

**Importance:**

Bone mass accrual is influenced by environmental and lifestyle factors. Targeted interventions at the early stages of life might decrease fracture and/or osteoporosis risk later in life.

**Objective:**

To investigate whether early-life exposure to residential surrounding green space is associated with a change in bone mineral density in young children.

**Design, Setting, and Participants:**

In this prospective birth cohort study (ENVIRONAGE [Environmental Influence on Aging in Early Life]), mother-child pairs from Flanders, Belgium, were recruited at birth and followed up for 4 to 6 years, between October 1, 2014, and July 31, 2021. Data analysis was conducted between January and February 2022.

**Exposures:**

Green space was estimated for high green (>3 m vegetation height), low green (≤3 m vegetation height ), and total green (sum of high and low) within several radii (100-3000 m) around the residence after geocoding of the addresses.

**Main Outcomes and Measures:**

Radial bone mineral density was assessed using quantitative ultrasound measurement at follow-up, measured as the mean of the axially transmitted speed of sound in meters per second. Multiple linear and logistic regression models were used while accounting for relevant covariates and potential confounders.

**Results:**

The study population comprised 327 children (180 [55.0%] female; mean [SD] age, 4.6 [0.4] years at the follow-up evaluation). Early-life exposure to residential green space was associated with increased childhood bone health. An IQR increment in total green (21.2%) and high green (19.9%) space within 500 m was associated with an increase of 27.38 m/s (95% CI, 9.63-45.13 m/s) and 25.30 m/s (95% CI, 7.93-42.68 m/s) in bone mineral density, respectively. Additionally, an IQR increase in total (25.2%) and high (23.2%) green space within 1000 m was associated with a 67% (odds ratio, 0.33; 95% CI, 0.17-0.61) and 61% (odds ratio, 0.39; 95% CI, 0.18-0.75) lower risk of having a bone density lower than the sex-specific 10th percentile (3567.6 m/s for girls and 3522.8 m/s for boys).

**Conclusions and Relevance:**

In this study of children aged 4 to 6 years, higher bone mineral density and a lower risk of having low bone density were associated with higher residential green space exposure during childhood. These findings highlight the importance of early-life exposure to residential green space on bone health during critical periods of growth and development, with long-term implications.

## Key Points

**Question:**

Is early-life exposure to residential surrounding green space associated with childhood bone health?

**Findings:**

In this birth cohort study of 327 children aged 4 to 6 years, participants exposed to more green space within a 300- to 3000-m radius around the residence had significantly higher bone mineral density. Additionally, higher green space within a 1000-m radius was significantly associated with reduced odds of having low bone density.

**Meaning:**

This study emphasizes the importance of early-life exposure to green space on children’s bone health during crucial periods of growth and development.

## Teaser

This cohort study investigates the association between early-life exposure to residential surrounding green space and bone mineral density in children aged 4 to 6 years in Belgium.

## Introduction

Bone mass, a composite of bone size and mineral density, is a key determinant of bone strength throughout life.^1^ Peak bone mass is achieved in early adulthood and depends on the bone mass accrual during skeletal growth and development.^1,2^ For this reason, suboptimal accrual at a young age is as crucial to the onset of osteoporosis as bone loss through aging.^1,2,3^ Hence, targeted interventions on bone mass accrual at the early stages of life may decrease fracture and/or osteoporosis risk later in life.^1,2,4^ In addition to the influence exerted by genetic factors, early-life physiologic, lifestyle (eg, nutrition and physical activity), and environmental factors may also play an important role in bone mass accrual.^2^

Several studies have reported the benefits of early-life green space exposure on neurocognitive^5,6,7,8,9,10,11,12^ and social-behavioral development,^13,14^ as well as on the mental^5,7,9,15^ and emotional well-being of children.^16^ Moreover, higher green space exposure during childhood has also been associated with lower body mass index,^6,17^ reduced risk of overweight or obesity,^5,6,17^ lower blood pressure,^18^ and higher physical activity.^5,6,16,17^ Despite increasing evidence about the health benefits of green space exposure, the available studies^19,20^ on the association with bone mineral density are scarce. One large population-based, epidemiologic study^19^ reported that living in a greener area was associated with higher bone strength in adults, suggesting that residential exposure to greenness may positively influence bone health. Conversely, a longitudinal study^20^ conducted among elderly citizens did not observe a protective effect of residential greenness on bone health. In this context, the aim of this study was to investigate the association between early-life exposure to residential surrounding green space and bone mineral density in children aged 4 to 6 years living in an affluent society.

## Methods

### Study Population

This study is part of the ongoing prospective Belgian birth cohort ENVIRONAGE (Environmental Influence on Aging in Early Life) in which mother-newborn pairs are recruited at the time of delivery at the East-Limburg Hospital in Genk (Belgium) from February 2010 onward. The procedure of eligibility, enrollment, data, and sample collection at birth is described in detail elsewhere.^21^ At 4 to 6 years after birth, mother-child pairs were contacted and invited to participate in a follow-up examination at Hasselt University (Belgium). At the follow-up visit, the mother signed a written informed consent before (sub)clinical investigations took place, and the children gave oral permission. Study protocols were approved by the ethical committees of Hasselt University and East-Limburg Hospital in Genk and conducted in compliance with the principles of the Declaration of Helsinki.^22^ This study was performed according to the Strengthening the Reporting of Observational Studies in Epidemiology (STROBE) reporting guidelines for observational studies.^23^

A participation flowchart with inclusion and exclusion criteria is given in eFigure 1 in Supplement 1. A total of 1492 participants were aged 4 to 6 years between October 1, 2014, and July 31, 2021, of whom 972 met the criteria for follow-up (could be contacted, had not moved out of the area, could communicate in Dutch, had not died, or were not seriously ill). In total, 541 mother-child pairs (55.7%) were followed up, of whom 397 did not move during the study period. Among these, radial bone mineral density was assessed in 327 children. This population is representative of the population of Flanders and is spatially distributed within the province of Limburg in Flanders, Belgium.^21^

### Data Collection

A semistructured questionnaire containing questions on sociodemographic, lifestyle, and health information of the mother and child was completed during the follow-up visit. Children’s ethnicity was categorized based on the native country of their grandparents as European when at least 2 grandparents were of European origin and non-European when this criterion was not met. Information on ethnicity was collected considering its potential role as a confounding factor stemming from genetic background as well as possible variations in nutrition or other environmental factors. Household passive smoke exposure was reported and grouped into 3 categories: no; yes, through 1 parent; and yes, through both parents. Children’s daily screen time was assessed as the mean number of hours per day a child was watching television, playing videogames, and using a tablet and was coded as less than 1 hour per day, 1 to 2 hours per day, and more than 2 hours per day. Data on vitamin supplementation (yes or no) and daily dairy products consumption, such as cheese, yogurt, and all types of milk (<1 or ≥1 servings per day), in the 3 months before the follow-up visit were also collected. Maternal educational level was categorized into low (no diploma), middle (high school diploma), and high (college or university degree). Furthermore, based on residential address, the participants were assigned to statistical sectors (the smallest units for which the Belgian National Institute of Statistics compiles information). The data resource was then used to obtain neighborhood median annual income (in Euros) based on the median annual household income data by sector (2017).

Body weight was assessed to the nearest 0.1 kg using a digital scale, and height was measured using a fixed stadiometer with a scale range of 0.5 cm. Body mass index was calculated as weight in kilograms divided by height in meters squared.

### Radial Bone Mineral Density Assessment

Bone mineral density is commonly used as a proxy measure of overall bone strength.^2^ Considering the young age of the participants and in the context of large-scale research studies, we used quantitative ultrasonography, a radiation-free technique, to assess bone mineral density, which has proven to be well correlated with dual-energy x-ray absorptiometry measurements.^24^ At the follow-up visit, bone mineral density was assessed in the children’s forearms using a densitometer (Sunlight MiniOmni Bone Densitometer) coupled with the Sunlight pediatric software, version 4.1.14 (BeamMed Inc). This quantitative ultrasonography method uses axial transmission technology based on the measurement of the speed of ultrasonic waves propagating along the bone. The mean of the axially transmitted speed of sound was obtained in meters per second after 3 consistent measurement cycles at the distal third of the radius while the child was seated with the left elbow lying on the table and the thumb facing up. The measurements were independent of room temperature, and a built-in system quality verification check of the probe and the device was performed daily.

### Residential Surrounding Green Space Exposure

The residential addresses of the children were geocoded. Green space was obtained based on the high-resolution (1 × 1 m) land cover data in the Green Map of Flanders 2012, generated by the Agency for Geographic Information Flanders, which classifies all nonagricultural vegetation as green spaces. The percentage of green space was calculated for high green (vegetation height >3 m), low green (vegetation height ≤3 m), and total green (sum of high and low green space) within several radii (100, 300, 500, 1000, and 3000 m) around the residence. All analyses were performed using Geographic Information System functions with ArcGIS software, version 10 (Esri Inc).

### Statistical Analysis

Analyses were conducted between January and February 2022. Study population characteristics are expressed as means (SDs) or numbers (percentages). The distribution of green space surrounding the residential address is presented as the mean (SD) and IQR percentages. The correlations between the different radii are shown as Pearson and Spearman correlation coefficients. Residential green space within the different radii showed moderate to very strong correlations for the total green space (*r* = 0.68-0.94) and high green space (*r* = 0.58-0.93). For low green space, the correlation between the different radii was less strong compared with total and high green space (*r* = 0.44-0.89) (eFigure 2 in Supplement 1).

Simple and multiple linear regression analyses were also performed to examine the association between the child’s bone mass and the determinants of bone mineral density (eTable 1 in Supplement 1). Estimates are presented as the difference (95% CI) in child bone mineral density. Moreover, multiple linear regression analyses (equation available in eMethods 1 in Supplement 1) were performed to investigate the association between residential green space exposure within different radii and children’s bone mineral density. We tested the potential effect modification of the child’s sex by adding an interaction term between residential green space exposure and the child’s sex on radial bone mineral density. Because these interactions were not statistically significant (eTable 2 in Supplement 1), we presented the results for girls and boys combined. All multiple linear regression models were adjusted for relevant covariates, which were selected based on the literature review, such as the child’s sex, age, weight, height, ethnicity, and maternal educational level.^25,26,27,28^ Estimates are presented as the difference (95% CI) in child bone mineral density for an IQR increment in the percentage of residential surrounding total, high, and low green space within a 100- to 3000-m buffer. In addition, several sensitivity analyses were performed. To assess the robustness of our findings, we adjusted the main model separately for the child’s daily screen time, vitamin supplementation, daily dairy products consumption, season, and neighborhood median annual income. Finally, we constructed a comprehensive model that included all previously mentioned variables (ie, child’s sex, age, weight, height, ethnicity, maternal educational level, child’s daily screen time, vitamin supplementation, daily dairy products consumption, season, and neighborhood median annual income).

Furthermore, we aimed to investigate the probability of having low bone mineral density (eMethods 2 in Supplement 1). For this reason, we dichotomized bone mineral density based on a sex-specific 10th percentile threshold within the total study population, which was different for girls and boys. Logistic regression analyses were used to investigate the association between residential green space exposure and the risk of having a bone mass lower than the sex-specific 10th percentile. We also tested the potential effect modification of the child’s sex by adding the interaction term between residential green space exposure and the child’s sex on the risk of low bone mineral density. Because these interactions were not statistically significant (eTable 3 in Supplement 1), the results for girls and boys were presented combined. Results are expressed as the odds ratio (OR) (95% CI) of low bone mineral density for an IQR increment in residential surrounding green space within a 100- to 3000-m buffer. All models were adjusted for the aforementioned covariates.

Data management and statistical analyses were performed using RStudio environment software, version 4.2.0 (Posit PBC). A 2-sided *P* < .05 was considered statistically significant.

## Results

### Study Population

A detailed characterization of the ENVIRONAGE participants (n = 327) is presented in Table 1. Children (180 [55.0%] girls and 147 [45.0%] boys) had a mean (SD) age of 4.6 (0.4) years, a mean (SD) weight of 18.5 (2.4) kg, and a mean (SD) height of 107.4 (4.9) cm at the follow-up visit. Most were of European descent (311 [95.1%]) and were not exposed to passive smoke within the household (211 [77.3%]). A total of 147 children (53.5%) spent 1 to 2 hours a day in front of a screen, whereas 104 (37.8%) spent less than 1 hour and 24 (8.7%) more than 2 hours. Furthermore, 109 (33.3%) took vitamin supplements, and most consumed at least 1 dairy product per day (244 [87.5%]). The mean (SD) radial bone mineral density assessed at follow-up was 3678.4 (116.1) m/s.

**Table 1.** Characteristics of the Study Population^a^

Characteristic	Finding (N = 327)
**Child**	
Sex	
Female	180 (55.0)
Male	147 (45.0)
Age, mean (SD), y	4.6 (0.4)
Weight, mean (SD), kg	18.5 (2.4)
Height, mean (SD), cm	107.4 (4.9)
Ethnicity	
European	311 (95.1)
Non-European	16 (4.9)
Household passive smoke exposure^b^	
No	211 (77.3)
Via 1 parent	42 (15.4)
Via both parents	20 (7.3)
Daily screen time, h/d^c^	
<1	104 (37.8)
1-2	147 (53.5)
>2	24 (8.7)
Vitamin supplementation	
Yes	109 (33.3)
No	218 (66.7)
Dairy products consumption, servings/d^d^	
<1	35 (12.5)
≥1	244 (87.5)
Season	
Winter	83 (25.4)
Spring	100 (30.6)
Summer	88 (26.9)
Autumn	56 (17.1)
Radial bone mineral density, mean (SD), m/s	3678.4 (116.1)
**Mother**	
Age at follow-up, mean (SD), y	35.7 (4.1)
BMI at follow-up, mean (SD)	25.0 (4.5)
Educational level	
Low	15 (4.6)
Middle	80 (24.5)
High	232 (70.9)
Neighborhood median annual income, mean (SD), €	26 487.2 (2965.2)

Abbreviation: BMI, body mass index (calculated as weight in kilograms divided by height in meters squared).

^a^ Data are presented as number (percentage) of study participants unless otherwise indicated.

^b^ Data available for 273 participants.

^c^ Data available for 275 participants.

^d^ Data available for 279 participants.

Mothers had a mean (SD) age of 35.7 (4.1) years and a body mass index of 25.0 (4.5) at the follow-up visit. Most (232 [70.9%]) were highly educated, and the median annual income of their neighborhood was a mean (SD) of €26 487.2 (€2965.2).

The distribution of green spaces surrounding the residential address is summarized in Table 2. The mean (SD) and IQR percentages are presented for total green as well as for high and low green within multiple radii (100-3000 m). Mean (SD) total green space ranged from 49.5% (14.0%) to 55.9% (16.8%), whereas high green ranged from 15.1% (12.7%) to 34.3% (14.8%) and low green from 21.6% (6.3%) to 34.4% (10.7%) within 100 to 3000 m radius around the residence.

**Table 2.** Distribution of Green Space Surrounding the Residential Address Within a 100- to 3000-m Radius

Radius, m	Total green space (sum of high and low vegetation height), %		High green space (>3 m vegetation height), %		Low green space (≤3 m vegetation height), %	
	Mean (SD)	IQR	Mean (SD)	IQR	Mean (SD)	IQR
100	49.5 (14.0)	39.0-60.6	15.1 (12.7)	6.5-20.0	34.4 (10.7)	26.7-42.1
300	51.3 (14.1)	41.5-61.3	20.4 (13.5)	10.1-27.4	30.9 (8.9)	25.3-36.3
500	51.9 (15.0)	41.0-62.2	23.6 (14.3)	12.2-32.1	28.3 (8.4)	22.5-33.4
1000	53.0 (16.1)	40.0-65.2	28.4 (14.9)	16.3-39.6	24.6 (7.4)	20.1-28.2
3000	55.9 (16.8)	43.0-69.5	34.3 (14.8)	22.2-44.3	21.6 (6.3)	17.1-26.6

The determinants of bone mineral density are presented in eTable 1 in Supplement 1. Bone mineral density increased with the child’s age (1-year increase: increase of 53.79 m/s; 95% CI, 24.63 to 82.95 m/s; *P* < .001) and height (1-cm increase: increase of 3.01 m/s; 95% CI, 0.47 to 5.55 m/s; *P* = .02). None of the other determinants, including the child’s sex, weight, ethnicity, daily screen time, vitamin supplementation, or daily dairy products consumption; season; maternal educational level; and neighborhood median annual income, were significantly associated with the child’s bone mass.

### Association Between Residential Green Space Exposure and Bone Mineral Density

**Figure 1.**
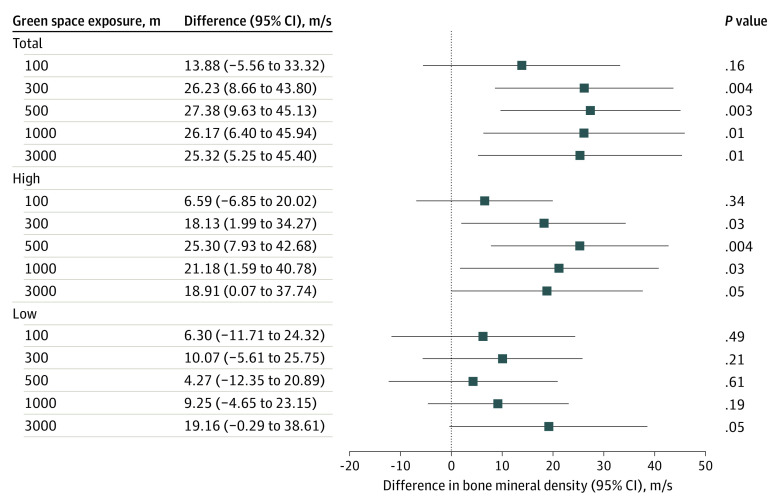
Association Between Child*’*s Bone Mineral Density and the Percentage of Residential Surrounding Green Space Within All Radii (100-3000 m) Estimates are presented as the difference (95% CI) in bone mineral density for an IQR increment in total green space (sum of high and low green), high green (>3 m), and low green (≤3 m) within a 100- to 3000-m radius. The model was adjusted for the child*’*s sex, ethnicity, age, weight, and height at follow-up and by maternal educational level (n = 327).

After adjustment for the selected covariates (child’s sex, age, weight, height, ethnicity, and maternal educational level), residential total green space exposure was positively associated with bone mineral density for all radii, with the exception of the closest buffer (100 m). The strongest association was observed for green space within a 500-m radius and indicated that an IQR increment in total green space (21.2%) was associated with a 27.38 m/s (95% CI, 9.63-45.13 m/s; *P* = .003) increase in bone mineral density (Figure 1). Comparable associations were found within the same radii for high green space with a vegetation height above 3 m. For instance, an IQR increase of 19.9% in high green space was associated with higher bone mineral density (25.30 m/s; 95% CI, 7.93-42.68 m/s; *P* = .004) within a 500-m radius. No significant associations were found regarding low green space with a vegetation height of 3 m or less.

**Figure 2.**
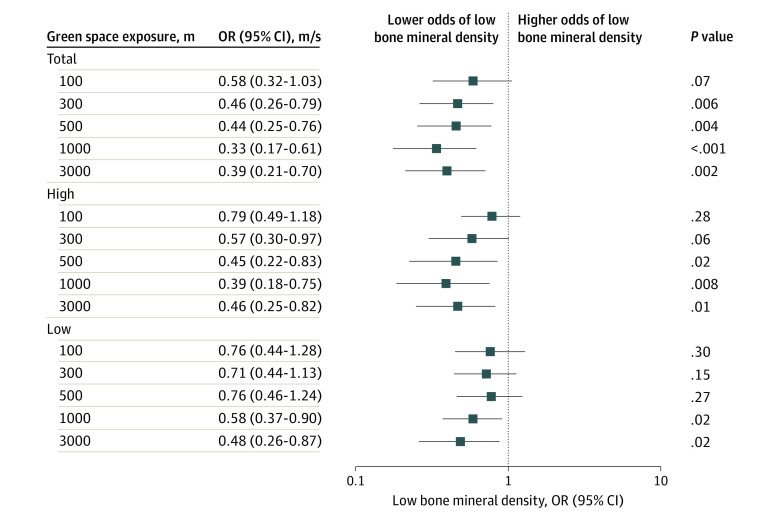
Association Between the Risk of Low Mineral Density and Percentage of Residential Surrounding Green Space for All Radii (100-3000 m) Estimates are presented as the odds ratio (OR) (95% CI) of low bone mineral density for an IQR increment in total green space (sum of high and low green vegetation height), high green (>3 m vegetation height), and low green (≤3 m vegetation height) within a 100- to 3000-m radius. The model was adjusted for the child’s sex, ethnicity, age, weight, and height at follow-up and by maternal educational level (n = 327).

In addition, we found that higher residential total and high green space exposures were significantly associated with a lower risk of having low bone mineral density within the same radii (Figure 2). Notably, an IQR (25.2%) increase in total green space within 1000 m was associated with a 67% lower risk (OR, 0.33; 95% CI, 0.17-0.61; *P* < .001) of having a bone mass lower than the sex-specific 10th percentile (3567.6 m/s for girls and 3522.8 m/s for boys). Likewise, an IQR (23.2%) increase in high green space within the same radii (1000 m) was associated with a 61% lower risk (OR, 0.39; 95% CI, 0.18-0.75; *P* = .008) of having a bone mass lower than the sex-specific 10th percentile. Significant associations were also found for low green space within the 1000- and 3000-m buffers.

### Sensitivity Analyses

The sensitivity analysis confirmed the robustness of these findings. The main model was adjusted for daily screen time (n = 275), vitamin supplementation (n = 327), daily dairy products consumption (n = 279), season (n = 327), and neighborhood median annual income (n = 327) separately (Table 3). All the observed associations between residential total green space exposure and bone mineral density remained significant. However, after adjustment for vitamin supplementation and daily dairy products consumption, the associations for high green exposure in the 3000-m buffer ceased to be significant. We also found a significant association between low green exposure in the 3000-m buffer and bone mineral density when adjusting for vitamin supplementation. Additionally, after the adjustment for all the variables in the full model, the associations for high green exposure in the 300- and 3000-m buffer ceased to be significant.

**Table 3.** Sensitivity Analysis for the Association Between Child’s Bone Mineral Density and the Percentage of Residential Surrounding Green Space Within All Radii (100-3000 m)^a^

Radius, m	Main model (n = 327)		Main model + daily screen time (n = 275)		Main model + vitamin supplementation (n = 327)		Main model + daily dairy products consumption (n = 279)		Main model + season (n = 327)		Main model + neighborhood annual median income (n = 327)		Full model (n = 262)	
	Difference (95% CI), m/s	*P* value	Difference (95% CI), m/s	*P* value	Difference (95% CI), m/s	*P* value	Difference (95% CI), m/s	*P* value	Difference (95% CI), m/s	*P* value	Difference (95% CI), m/s	*P* value	Difference (95% CI), m/s	*P* value
**Total green**														
100	13.88 (−5.56 to 33.32)	.16	12.90 (−8.28 to 34.09)	.23	13.50 (−5.97 to 32.98)	.17	18.56 (−2.15 to 39.26)	.08	15.01 (−4.83 to 34.85)	.14	15.50 (−4.25 to 35.25)	.12	20.47 (−4.45 to 45.39)	.11
300	26.23 (8.66 to 43.80)	.004	31.78 (11.81 to 51.74)	.002	26.05 (8.46 to 43.64)	.004	29.86 (10.36 to 49.36)	.003	26.52 (8.80 to 44.24)	.003	27.18 (9.51 to 44.86)	.003	31.98 (9.47 to 54.49)	.006
500	27.38 (9.63 to 45.13)	.003	33.78 (13.97 to 53.59)	.001	27.39 (9.64 to 45.15)	.003	30.18 (11.14 to 49.22)	.002	27.54 (9.68 to 45.41)	.003	28.05 (10.24 to 45.86)	.002	34.67 (12.28 to 57.05)	.003
1000	26.17 (6.40 to 45.94)	.01	34.41 (11.55 to 57.26)	.003	26.11 (6.32 to 45.90)	.01	28.97 (7.40 to 50.54)	.009	26.36 (6.47 to 46.26)	.01	26.60 (6.79 to 46.42)	.009	33.85 (9.87 to 57.83)	.006
3000	25.32 (5.25 to 45.40)	.01	29.22 (5.84 to 52.61)	.01	25.15 (5.05 to 45.24)	.01	25.80 (3.70 to 47.89)	.02	25.66 (5.42 to 45.90)	.01	25.38 (5.29 to 45.47)	.01	28.37 (4.15 to 52.58)	.02
**High green (>3 m)**														
100	6.59 (−6.85 to 20.02)	.34	7.72 (−7.76 to 23.20)	.33	6.31 (−7.15 to 19.77)	.36	9.63 (−4.71 to 23.98)	.19	7.18 (−6.48 to 20.84)	.30	6.87 (−6.60 to 20.34)	.32	9.86 (−6.94 to 26.67)	.25
300	18.13 (1.99 to 34.27)	.03	18.82 (0.24 to 37.39)	.05	17.79 (1.61 to 33.98)	.03	19.27 (0.96 to 37.57)	.04	18.51 (2.25 to 34.78)	.03	18.72 (2.51 to 34.93)	.02	17.66 (−2.48 to 37.79)	.09
500	25.30 (7.93 to 42.68)	.004	28.11 (8.46 to 47.77)	.005	25.01 (7.60 to 42.42)	.005	26.92 (7.83 to 46.01)	.006	25.58 (8.09 to 43.06)	.004	25.96 (8.52 to 43.40)	.004	28.85 (7.25 to 50.45)	.009
1000	21.18 (1.59 to 40.78)	.03	26.16 (3.03 to 49.30)	.03	20.86 (1.23 to 40.48)	.04	22.82 (0.84 to 44.80)	.04	21.51 (1.79 to 41.23)	.03	21.88 (2.22 to 41.55)	.03	25.71 (1.63 to 49.79)	.04
3000	18.91 (0.07 to 37.74)	.05	22.25 (0.11 to 44.39)	.05	18.47 (−0.42 to 37.36)	.06	18.86 (−2.22 to 39.94)	.08	19.38 (0.37 to 38.38)	.05	19.20 (0.34 to 38.06)	.05	21.57 (−1.31 to 44.45)	.06
**Low green (≤3 m)**														
100	6.30 (−11.71 to 24.32)	.49	3.80 (−16.6 to 24.20)	.71	6.27 (−11.76 to 24.29)	.49	7.40 (−12.43 to 27.24)	.46	6.44 (−11.67 to 24.56)	.48	7.44 (−10.81 to 25.70)	.42	7.42 (−14.56 to 29.39)	.51
300	10.07 (−5.61 to 25.75)	.21	16.65 (−1.74 to 35.04)	.08	10.39 (−5.31 to 26.09)	.19	14.02 (−3.66 to 31.70)	.12	9.87 (−5.96 to 25.7)	.22	10.31 (−5.40 to 26.01)	.20	16.66 (−3.15 to 36.47)	.10
500	4.27 (−12.35 to 20.89)	.61	9.59 (−9.12 to 28.30)	.31	4.88 (−11.80 to 21.57)	.56	7.73 (−12.06 to 27.52)	.44	4.06 (−12.68 to 20.81)	.63	4.29 (−12.35 to 20.92)	.61	8.54 (−12.12 to 29.21)	.42
1000	9.25 (−4.65 to 23.15)	.19	14.99 (−0.90 to 30.87)	.06	9.74 (−4.21 to 23.68)	.17	13.34 (−3.57 to 30.25)	.12	9.11 (−4.89 to 23.11)	.20	9.03 (−4.91 to 22.96)	.20	14.79 (−1.92 to 31.51)	.08
3000	19.16 (−0.29 to 38.61)	.05	22.46 (−0.99 to 45.90)	.06	20.11 (0.57 to 39.65)	.04	22.20 (−0.42 to 44.83)	.05	18.91 (−0.68 to 38.51)	.06	18.77 (−0.76 to 38.31)	.06	21.10 (−2.33 to 44.54)	.08

^a^ Estimates are presented as the difference (95% CI) in bone mineral density for an IQR increment in total green space (sum of high and low green), high green (>3 m), and low green (≤3 m) within a 100- to 3000-m radius. The main model was adjusted for the child’s sex, ethnicity, age, weight, and height at follow-up and by maternal educational level.

## Discussion

In this prospective birth cohort study, we investigated whether early-life exposure to residential surrounding green space was associated with bone mineral density. The key findings of our analysis were that higher residential green space exposure was associated with increased bone mineral density as well as with a lower risk of having a low bone mass in children aged 4 to 6 years.

To the best of our knowledge, this is the first study to assess the association between residential green space exposure and bone health in children. Our results showed that an increment in residential total (sum of high and low green) and high green (vegetation height >3 m) space was associated with increased bone mineral density within a 500-m radius. In comparison, 2 studies^19,20^ were recently conducted in adults. Jiang and colleagues^19^ reported similar results within a cross-sectional study enrolling 66 053 adults aged 30 to 79 years recruited from southwest China. A positive association was outlined between residential exposure to greenness, within 500-m and 1000-m radii, and heel bone strength.^19^ On the other hand, within a prospective cohort study of 3944 adults aged 65 years or older from Hong Kong, Lin et al^20^ observed no convincing associations between greenness and changes in bone mineral density of the total hip, femoral neck, and whole body within 300-m and 500-m radii.^20^ The deviation from our findings may be explained by the divergence in study design and participant age but mostly by the existing differences in living and built environments. Hong Kong is a very densely populated city, with most of its 7 million inhabitants living in medium- or high-density areas, whereas most green spaces are located in rural areas, which are not easily accessible to urban residents.^20^ In contrast, our study population lived in a region that combines urban, suburban, and rural areas, with population densities ranging from 82 to 743 inhabitants per kilometer,^21^ and our participants were, on average, exposed to 52% of total green space, within a 500-m radius surrounding their residential address.

The beneficial impact of surrounding green space within a 500-m radius is in line with previous research into health-related effects of green space exposure in children.^18^ Notably, a 500-m buffer represents a distance that is reachable within a 10-minute walk.^18,29^ In this study, no significant associations between the child’s radial bone density and the closest buffer (100 m) were observed. Accordingly, the availability of accessible green spaces within walking distance from home, their safety, and the diversity of natural and social facilities have been reported to impact green space use.^30^ Thus, it is plausible that our participants were more likely to walk from home to the nearest available and accessible green space (eg, park, garden, or forest) within 500 to 1000 m around the residence.

Increasing physical activity has been hypothesized to be one of the pathways underlying the beneficial health effects of green space exposure.^31^ The mechanical loading caused by physical activity can activate signaling pathways that trigger the activity of osteoblast bone formation and osteoclast bone resorption, leading to substantial and consistent positive effects on bone development.^32,33,34^ Beneficial influences on bone mass accrual during childhood and adolescence are mainly documented for moderate-to-vigorous-intensity physical activities, especially weight-bearing aerobic activities (eg, hiking, skipping, and jogging) that are commonly practiced in green spaces.^32,34,35^ In line with this, previous studies^36,37,38,39^ conducted in youth populations showed positive associations between neighborhood green space and physical activity. In addition to physical activity, screen time in young children might be impacted by neighborhood green space levels.^40^ In this study, data on the child’s screen time were used as a proxy of sedentary behavior, although we could not observe an association between screen time and surrounding residential green space.

### Strengths and Limitations

This study has several strengths. First, we provided novel evidence about the positive effects of long-term residential green space exposure on bone mineral density in young children. Second, multiple sensitivity analyses confirmed the robustness of our findings. Third, we used high-resolution green exposure data with detailed information on the surrounding type of green, which might contribute to understanding the mechanisms underlying the beneficial effects of green space exposure on health outcomes in children.^41^ Fourth, the present findings are unlikely to be a product of multiple comparisons, because we tested an a priori hypothesis involving bone mineral density and strongly correlated green space indicators, of which each buffer is not independent of the others. Therefore, the green space variables did not provide an independent opportunity for a type I error. For this reason, we did not adjust for multiple testing. Nevertheless, it is unlikely that our findings are only a reflection of chance. In fact, consistent associations between children’s bone mineral density and residential surrounding total and high green space exposure were observed within a 300- to 3000-m radius.

However, some limitations should be acknowledged. First, because no detailed information about the frequency and duration of indoor and/or outdoor physical activity was available, we used data on children’s screen time as a proxy of sedentary behavior. Second, no data about the children’s use of surrounding residential green spaces, as well as accessibility and safety, were available. Third, bone mineral density was only assessed on the forearm using quantitative ultrasound measurements. Nevertheless, this radiation-free technique has proven to be appropriate for screening in children and is well correlated with dual-energy x-ray absorptiometry, which is used in clinical settings.^24^

## Conclusions

This prospective birth cohort study provides evidence about the importance of the surrounding residential green space from birth until age 4 to 6 years on bone health. Our findings are of public health relevance because they contribute to disentangle the complex associations between health and the characteristics of the built environment. Moreover, this study highlights the urgent need to raise awareness among policy makers on the importance of conserving and expanding residential green spaces to maximize bone mineral density during crucial periods of growth and development. The promotion of such preventive strategies might decrease fracture and/or osteoporosis risk later in life, resulting in financial, physical, and psychological benefits for the individual and the community.

## Notes

Supplement 1. **eMethods 1.** Multiple Linear Regression Equation **eMethods 2. **Logistic Regression Equation **eTable 1.** Determinants of Bone Mineral Density Presented as the Difference (95% CI) in m/s **eTable 2.** Association Between Child's Bone Mineral Density and the Percentage of Residential Surrounding Green Space Within All Radii (100-3000 m) for the Total Population (N = 327), Girls (n = 180), and Boys (n = 147) **eTable 3.** Association Between the Risk of Low Mineral Density and Percentage of Residential Surrounding Green Space Within All Radii (100-3000 m) for the Total Population (N = 327), Girls (n = 180), and Boys (n = 147) **eFigure 1.** Flowchart Describing the Included ENVIRONAGE Participants **eFigure 2.** Pearson (Left) and Spearman (Right) Correlation Matrix Between Residential Green Space Radius (100-3000 m) for Total Green (Sum of High and Low Green), High Green (>3 m), and Low Green (≤3 m)

Supplement 2.
Data Sharing Statement
