# Supplementary material for: Exposure to Residential Green Space and Bone Mineral Density in Young Children
Source: JAMA Netw Open. 2024 Jan 4;7(1):e2350214. doi: 10.1001/jamanetworkopen.2023.50214 (PMC10767584; doi:10.1001/jamanetworkopen.2023.50214)
Supplement: Supplement 1. — eMethods 1. Multiple Linear Regression Equation eMethods 2. Logistic Regression Equation eTable 1. Determinants of Bone Mineral Density Presented as the Difference (95% CI) in m/s eTable 2. Association Between Child's Bone Mineral Density and the Percentage of Residential Surrounding Green Space Within All Radii (100-3000 m) for the Total Population (N = 327), Girls (n = 180), and Boys (n = 147) eTable 3. Association Between the Risk of Low Mineral Density and Percentage of Residential Surrounding Green Space Within All Radii (100-3000 m) for the Total Population (N = 327), Girls (n = 180), and Boys (n = 147) eFigure 1. Flowchart Describing the Included ENVIRONAGE Participants eFigure 2. Pearson (Left) and Spearman (Right) Correlation Matrix Between Residential Green Space Radius (100-3000 m) for Total Green (Sum of High and Low Green), High Green (>3 m), and Low Green (≤3 m) [file jamanetwopen-e2350214-s001.pdf]

Supplementary Online Content

Sleurs H, Silva AI, Bijnens EM, et al. Exposure to residential green space and bone mineral density in young children. *JAMA Netw Open*. 2024;7(1):e2350214. doi:10.1001/jamanetworkopen.2023.50214

eMethods 1. Multiple Linear Regression Equation

eMethods 2. Logistic Regression Equation

eTable 1. Determinants of Bone Mineral Density Presented as the Difference (95% CI) in m/s

eTable 2. Association Between Child's Bone Mineral Density and the Percentage of Residential Surrounding Green Space Within All Radii (100-3000 m) for the Total Population (N=327), Girls (n=180), and Boys (n=147)

eTable 3. Association Between the Risk of Low Mineral Density and Percentage of Residential Surrounding Green Space Within All Radii (100-3000 m) for the Total Population (N=327), Girls (n=180), and Boys (n=147)

eFigure 1. Flowchart Describing the Included ENVIRONAGE Participants

eFigure 2. Pearson (Left) and Spearman (Right) Correlation Matrix Between Residential Green Space Radius (100-3000 m) for Total Green (Sum of High and Low Green), High Green (> 3 m), and Low Green (≤ 3 m)

This supplementary material has been provided by the authors to give readers additional information about their work.

© 2024 Sleurs H et al. *JAMA Network Open.*

eMethods 1. Multiple linear regression equation

$$Y_i = \beta_0 + \beta_1 X_{i1} + \beta_2 X_{i2} + \ldots + \beta_p X_{ip} + \varepsilon_i$$

Where Y_i is the child's bone mineral density for the i-th observation, β_0 corresponds to the intercept term, $\beta_{1..p}$ denotes the regression coefficient associated with each independent variable, $X_{i1 \ldots ip}$ represents the p independent variables for the i-th observation (green space exposure, child's sex, age, weight, height, ethnicity, maternal education, child's daily screen time, vitamin supplementation, daily dairy products consumption, season, and neighborhood median annual income), and ε_i corresponds to the error term for the i-th observation.

© 2024 Sleurs H et al. *JAMA Network Open.*

eMethods 2. Logistic regression equation

When the bone mineral density is coded as a binary outcome, where $Y_i = 0$ when the i-th child's bone mineral density is higher or equal to the sex-specific 10th percentile of the study population, and $Y_i = 1$ when the i-th child's bone mineral density is lower than the sex-specific 10th percentile of the study population, the probability of having bone mineral density lower than the sex-specific 10th percentile of the study population is denoted as $\pi_i = P(Y_i = 1)$. This probability, π_i, is connected with the linear predictor via a logit link function:

$$\log\left(\frac{\pi_i}{1-\pi_i}\right) = \beta_0 + \beta_1 X_{i1} + \beta_2 X_{i2} + \ldots + \beta_p X_{ip} + \varepsilon_i$$

where β_0 is the intercept term, $\beta_{1..p}$ corresponds to the regression coefficient associated with each independent variable, $X_{i1\ldots ip}$ represents the p independent variables for the i-th observation (green space exposure, child's sex, age, weight, height, ethnicity, maternal education), and ε_i denotes the error term for the i-th observation.

© 2024 Sleurs H et al. *JAMA Network Open.*

eTable 1. Determinants of bone mineral density presented as the difference (95% CI) in m/s

	Simple regression		Multiple regression	
	Difference (95% CI), m/s	Pvalue	Difference (95% CI), m/s	P value
**Child**				
Sex, girls	7.34 (-17.99 to 32.67)	0.57	2.38 (-27.53 to 32.29)	0.88
Age, +1 y	53.79 (24.63 to 82.95)	<0.001	45.69 (-2.04 to 93.42)	0.06
Weight, +1 kg	4.75 (-0.43 to 9.93)	0.07	-1.64 (-12.01 to 8.74)	0.76
Height, +1 cm	3.01 (0.47 to 5.55)	0.02	-0.01 (-5.58 to 5.55)	1.00
Ethnicity, Non-European	30.70 (-27.64 to 89.03)	0.30	18.24 (-52.93 to 89.41)	0.62
Daily screen time, h/d				
< 1	Reference		Reference	
1-2	12.87 (-17.37 to 43.11)	0.41	15.64 (-16.03 to 47.31)	0.33
> 2	15.99 (-37.48 to 69.46)	0.56	9.20 (-47.48 to 65.88)	0.75
Vitamin supplementation, Yes	-14.45 (-41.14 to 12.24)	0.29	-24.33 (-55.45 to 6.79)	0.13
Daily dairy products consumption, serving/day	5.27 (-8.52 to 19.06)	0.46	4.79 (-9.95 to 19.54)	0.52
Season				
Winter	Reference		Reference	
Spring	-1.44 (-35.35 to 32.47)	0.93	-16.37 (-57.12 to 24.38)	0.43
Summer	-11.76 (-46.69 to 23.18)	0.51	-22.81 (-63.23 to 17.61)	0.27
Autumn	-13.54 (-53.03 to 25.95)	0.50	-1.57 (-49.15 to 46.02)	0.95
**Mother**				
Education level				
Low	Reference		Reference	
Middle	26.48 (-37.63 to 90.59)	0.42	33.42 (-38.18 to 105.02)	0.36
High	32.45 (-28.25 to 93.15)	0.30	52.70 (-15.47 to 120.87)	0.13
Neighborhood median annual income, +1000 €	-2.13 (-6.38 to 2.12)	0.33	-3.80 (-9.29 to 1.69)	0.18

© 2024 Sleurs H et al. *JAMA Network Open.*

eTable 2. Association between child's bone mineral density and the percentage of residential surrounding green space within all radii (100-3000 m) for the total population (n=327), girls (n=180) and boys (n=147)

	Total population (n=327)		Girls (n=180)		Boys (n=147)		
	**Difference (95% CI), m/s**	**P value**	**Difference (95% CI), m/s**	**P value**	**Difference (95% CI), m/s**	**P value**	**P value for interaction[a]**
**Total green**							
100 m	13.88 (-5.56 to 33.32)	0.16	2.21 (-19.28 to 23.69)	0.84	33.58 (-0.91 to 68.07)	0.06	0.16
300 m	26.23 (8.66 to 43.80)	0.004	14.96 (-3.71 to 33.64)	0.12	44.89 (11.24 to 78.54)	0.009	0.25
500 m	27.38 (9.63 to 45.13)	0.003	20.42 (-0.52 to 41.36)	0.06	38.42 (7.79 to 69.05)	0.01	0.46
1000 m	26.17 (6.40 to 45.94)	0.01	19.55 (-2.67 to 41.77)	0.08	37.65 (2.52 to 72.77)	0.04	0.53
3000 m	25.32 (5.25 to 45.40)	0.01	14.77 (-10.29 to 39.82)	0.25	38.32 (6.15 to 70.48)	0.02	0.25
**High green (>3 m)**							
100 m	6.59 (-6.85 to 20.02)	0.34	2.33 (-14.98 to 19.65)	0.79	11.75 (-6.38 to 29.88)	0.20	0.41
300 m	18.13 (1.99 to 34.27)	0.03	11.53 (-7.36 to 30.43)	0.23	27.67 (0.4 to 54.95)	0.05	0.41
500 m	25.30 (7.93 to 42.68)	0.004	16.02 (-3.79 to 35.83)	0.11	38.24 (8.53 to 67.96)	0.01	0.33
1000 m	21.18 (1.59 to 40.78)	0.03	16.11 (-6.02 to 38.24)	0.15	33.74 (-2.28 to 69.75)	0.07	0.51
3000 m	18.91 (0.07 to 37.74)	0.05	8.11 (-14.07 to 30.3)	0.47	34.29 (2.19 to 66.39)	0.04	0.20
**Low green (≤3 m)**							
100 m	6.30 (-11.71 to 24.32)	0.49	-0.61 (-22.08 to 20.85)	0.96	17.35 (-14.26 to 48.95)	0.28	0.31
300 m	10.07 (-5.61 to 25.75)	0.21	6.62 (-12.62 to 25.85)	0.50	13.88 (-12.56 to 40.33)	0.30	0.60
500 m	4.27 (-12.35 to 20.89)	0.61	7.02 (-14.05 to 28.08)	0.51	0.62 (-27.62 to 28.85)	0.97	0.73
1000 m	9.25 (-4.65 to 23.15)	0.19	6.04 (-11.07 to 23.15)	0.49	12.81 (-11.45 to 37.06)	0.30	0.60
3000 m	19.16 (-0.29 to 38.61)	0.05	16.21 (-7.69 to 40.12)	0.18	22.71 (-9.18 to 54.6)	0.16	0.73

Estimates are presented as the difference (95% CI) in bone mineral density (m/s) for an interquartile range (IQR) increment in total green space (sum of high and low green), high green (> 3 m) and low green (≤ 3 m) within a 100-3000 m radius for the total population (n= 327), girls (n= 180) and boys (n= 147). The main model was adjusted for the child's sex, ethnicity, age, weight, and height at follow-up and by maternal education. [a]Represents the overall p-value for the interaction green space x child's sex.

© 2024 Sleurs H et al. *JAMA Network Open.*

eTable 3. Association between the risk of low mineral density and percentage of residential surrounding green space within all radii (100-3000 m) for the total population (n=327), girls (n=180), and boys (n=147)

	Total population (n=327)		Girls (n=180)		Boys (n=147)		
	**OR (95% CI), m/s**	**P value**	**OR (95% CI), m/s**	**pPva lue**	**OR (95% CI), m/s**	**Pvalu e**	**P value for interaction[a]**
**Total green**							
100 m	0.58 (0.32 to 1.03)	0.07	0.92 (0.40 to 2.06)	0.85	0.34 (0.13 to 0.8)	0.02	0.14
300 m	0.46 (0.26 to 0.79)	0.006	0.51 (0.25 to 1.00)	0.06	0.37 (0.13 to 0.9)	0.04	0.83
500 m	0.44 (0.25 to 0.76)	0.004	0.38 (0.17 to 0.81)	0.01	0.48 (0.20 to 1.07)	0.08	0.62
1000 m	0.33 (0.17 to 0.61)	<0.001	0.29 (0.11 to 0.64)	0.004	0.36 (0.14 to 0.88)	0.03	0.63
3000 m	0.39 (0.21 to 0.70)	0.002	0.36 (0.15 to 0.83)	0.02	0.41 (0.17 to 0.90)	0.03	0.94
**High green (>3 m)**							
100 m	0.79 (0.49 to 1.18)	0.28	0.85 (0.41 to 1.53)	0.61	0.67 (0.32 to 1.15)	0.21	0.46
300 m	0.57 (0.30 to 0.97)	0.06	0.52 (0.20 to 1.08)	0.12	0.57 (0.21 to 1.25)	0.21	0.84
500 m	0.45 (0.22 to 0.83)	0.02	0.35 (0.12 to 0.82)	0.03	0.55 (0.20 to 1.28)	0.20	0.44
1000 m	0.39 (0.18 to 0.75)	0.008	0.27 (0.09 to 0.68)	0.01	0.5 (0.17 to 1.27)	0.17	0.39
3000 m	0.46 (0.25 to 0.82)	0.01	0.41 (0.17 to 0.90)	0.03	0.49 (0.20 to 1.12)	0.10	0.83
**Low green (≤3 m)**							
100 m	0.76 (0.44 to 1.28)	0.30	1.17 (0.53 to 2.63)	0.70	0.47 (0.20 to 1.03)	0.07	0.14
300 m	0.71 (0.44 to 1.13)	0.15	0.82 (0.42 to 1.63)	0.57	0.6 (0.29 to 1.17)	0.15	0.54
500 m	0.76 (0.46 to 1.24)	0.27	0.8 (0.37 to 1.70)	0.56	0.67 (0.32 to 1.36)	0.28	0.76
1000 m	0.58 (0.37 to 0.90)	0.02	0.68 (0.34 to 1.26)	0.24	0.44 (0.21 to 0.85)	0.02	0.37
3000 m	0.48 (0.26 to 0.87)	0.02	0.57 (0.23 to 1.30)	0.19	0.37 (0.15 to 0.86)	0.03	0.36

Estimates are presented as the odds ratio (OR) (95% CI) of low bone mineral density for an interquartile range (IQR) increment in total green space (sum of high and low green) within a 100-3000 m radius for the total population (n= 327), girls (n= 180) and boys (n= 147). The model was adjusted for the child's sex, ethnicity, age, weight, and height at follow-up and by maternal education (n= 327). [a]Represents the overall p-value for the interaction green space x child's sex.

© 2024 Sleurs H et al. *JAMA Network Open.*

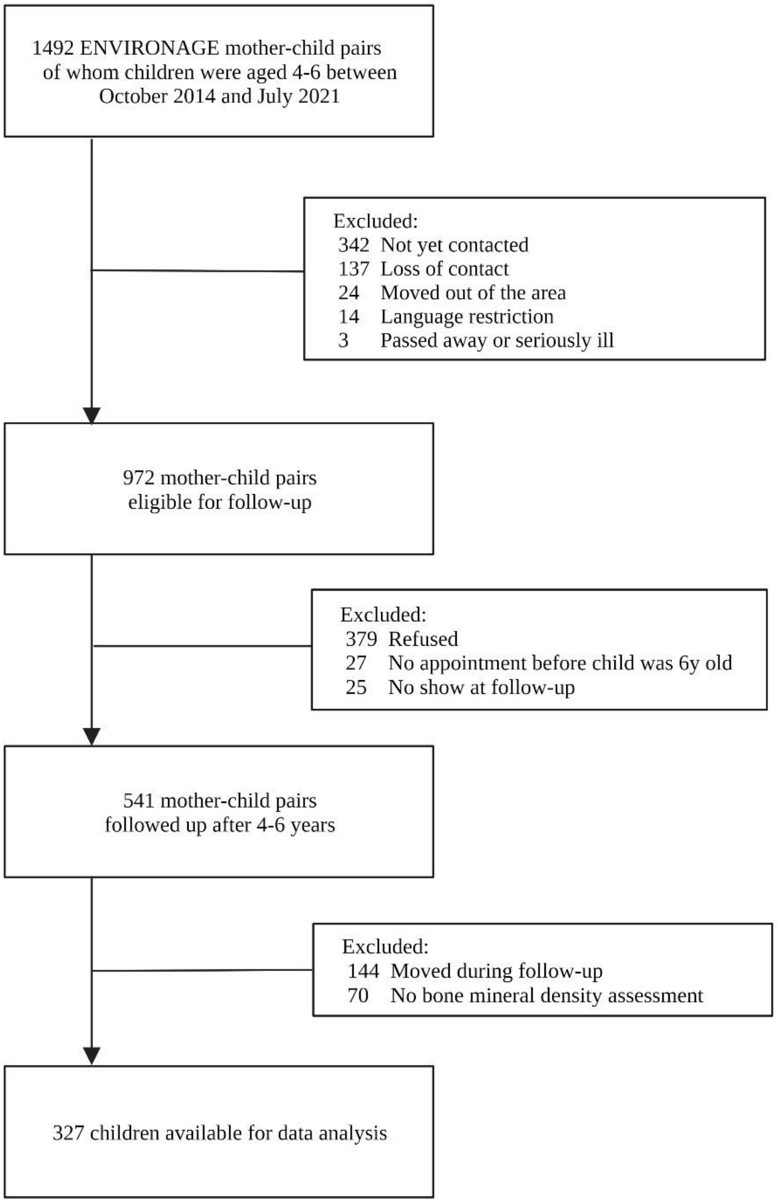

eFigure 1. Flowchart describing the included ENVIRONAGE participants

© 2024 Sleurs H et al. *JAMA Network Open.*

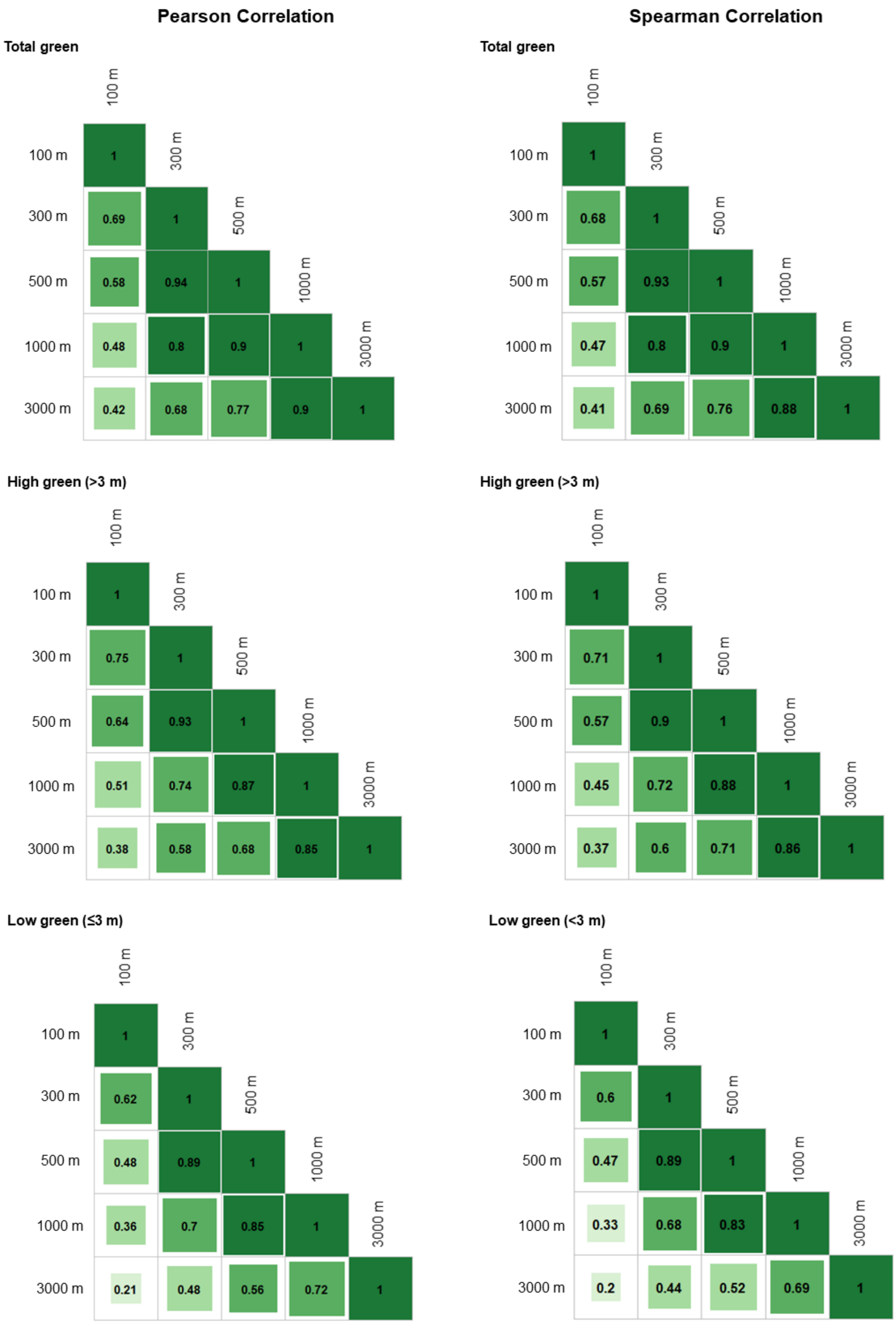

© 2024 Sleurs H et al. *JAMA Network Open.*

eFigure 2. Pearson (left) and Spearman (right) correlation matrix between residential green space radius (100-3000 m) for total green (sum of high and low green), high green (>3 m), and low green (≤3 m)

© 2024 Sleurs H et al. *JAMA Network Open.*
