# Supplementary material for: Exposure to Residential Green Space and Bone Mineral Density in Young Children
Source: JAMA Netw Open. 2024 Jan 4;7(1):e2350214. doi: 10.1001/jamanetworkopen.2023.50214 (PMC10767584; doi:10.1001/jamanetworkopen.2023.50214)
Supplement: Supplement 2. — Data Sharing Statement [file jamanetwopen-e2350214-s002.pdf]

Data Sharing Statement

Sleurs. Exposure to Residential Green Space and Bone Mineral Density in Young Children. *JAMA Netw Open*. Published January 04, 2024. doi:10.1001/jamanetworkopen.2023.50214

Data
Data available: No

Additional Information
Explanation for why data not available: Data can be requested by email to corresponding author
